# Repeated Treatments with Ingenol Mebutate Prevents Progression of UV-Induced Photodamage in Hairless Mice

**DOI:** 10.1371/journal.pone.0162597

**Published:** 2016-09-16

**Authors:** Andrés Már Erlendsson, Daniel Thaysen-Petersen, Christiane Bay, Andreas Hald, Kresten Skak, John Robert Zibert, Uwe Paasch, Hans Christian Wulf, Merete Haedersdal

**Affiliations:** 1 Department of Dermatology, Bispebjerg University Hospital, Copenhagen, Denmark; 2 Leo Pharma A/S, Ballerup, Denmark; 3 Department of Dermatology, Division of Aesthetics and Laserdermatology, University of Leipzig, Leipzig, Germany; Ohio State University, UNITED STATES

## Abstract

**Background and Aim:**

Ingenol mebutate (IngMeb) is an effective treatment for actinic keratosis. In this study, we hypothesized that repeated treatments with IngMeb may prevent progression of UV-induced photodamage, and that concurrent application of a corticosteroid may reduce IngMeb-induced local skin responses (LSR).

**Methods:**

Hairless mice (n = 60; 3 groups of 20 mice) were irradiated with solar simulated ultraviolet radiation (UVR) throughout the study. Five single treatments with IngMeb were given at 4-week intervals (Days 21, 49, 77, 105, and 133). Clobetasol propionate (CP) was applied once daily for 5 days prior to each IngMeb application, as well as 6 h and 1 day post treatment. One week after IngMeb treatment No. 1, 3, and 5 (Days 28, 84, and 140), biopsies from four mice in each group were collected for histological evaluation of UV-damage on a standardized UV-damage scale (0–12). LSR (0–24) were assessed once daily (Days 1–7) after each IngMeb treatment.

**Results:**

IngMeb prevented progression of photodamage in terms of keratosis grade, epidermal hypertrophy, dysplasia, and dermal actinic damage with a lower composite UV-damage score on day 140 (UVR 10.25 vs. UVR+IngMeb 6.00, p = 0.002) compared to UVR alone. IngMeb induced LSR, including erythema, flaking, crusting, bleeding, vesiculation, and ulceration. Concurrent CP increased LSR (max LSR Tx 1–5: UVR+IngMeb+CP 3.6–5.5 vs. UVR+IngMeb 2.6–4.3) and provided better prevention of photodamage compared to IngMeb alone (Day 140: UVR+IngMeb 6.00 vs. UVR+IngMeb+CP 3.00 p < 0.001).

**Conclusion:**

Repeated field-directed treatments with IngMeb prevent progression of cutaneous photodamage in hairless mice, while CP cannot be used to alleviate IngMeb-induced LSR. The findings suggest that IngMeb may potentially serve as a prophylactic treatment for UV-induced tumors.

## Introduction

Non-melanoma skin cancer (NMSC) is the most commonly diagnosed cancer globally and is primarily constituted by basal cell carcinomas (BCC) and squamous cell carcinomas (SCC) [1]. Ultraviolet radiation (UVR) is accoun for 90% of BCC and SCC and acts as a complete carcinogen capable of tumor initiation, promotion, and progression [2].

UVB (290–320 nm) is directly absorbed by DNA bases, producing photolesions such as cyclobutane pyrimidine dimers (CPD) and 6–4 photoproducts [3]. If not repaired, these photolesions cause signature UV mutations i.e. ‘C to T’ or ‘CC to TT’^3^. In addition, UVR (UVA 320–400 nm and UVB), absorbed by various skin chromophores, may result in production of reactive oxygen species (ROSs) [4]. Through oxidative stress, ROSs are believed to cause indirect DNA damage, and may play a role in tumor promotion [5]. Through continuous oxidative stress and accumulation of mutations, processes like proliferation, differentiation, and apoptosis fail; unresponsive to normal cell-signaling pathways, aberrant cells may grow into dysplastic colonies and eventually undergo malignant progression [6].

Ingenol mebutate (IngMeb) is a topical drug, approved for field-treatment of actinic keratoses (AKs) [7]. IngMeb induces loss of plasma and mitochondrial membrane potential in keratinocytes, facilitating Ca^2+^ influx, which results in swelling and induction of cell death by necrosis and apoptosis [8,9]. IngMeb also activates various Protein Kinase C (PKC) isomers, especially PKC-δ, which is suggested to induce apoptosis [10,11], while also increasing innate-immune surveillance by neutrophil recruitment [10–14]. In a murine study, application of IngMeb to photodamaged skin resulted in a 70% reduction of p53-mutated patches as well as a 70% reduction in the number of tumors that subsequently emerged [15]. As IngMeb is successful in reversing pre-existing actinic damage, repeated treatments with IngMeb may hold promise in preventing progression of UV-induced photodamage.

A prerequisite for prophylactic treatments is minimal side effects. IngMeb is however known to induce acute inflammation of varying intensity and minimizing the inflammation is of importance before IngMeb can be considered for prophylactic purposes [7,16]. Clobetasol propionate (CP) is a potent glucocorticoid with immunosuppressive, anti-inflammatory, and vasoconstrictive properties [17]. Topical application of dexamethasone has previously been found to prevent IngMeb-induced neutrophil invasion, and CP may similarly be used to alleviate IngMeb-induced inflammation [18].

In this study, we hypothesized that repeated treatments with IngMeb may prevent progression of UV damage, and that application of a corticosteroid may reduce IngMeb-induced local skin responses (LSR).

## Methods

### Animals

A total of 60 hairless, immunocompetent C3.Cg/TifBomTac mice of female gender (Taconic, Lille Skensved, Denmark) were randomized to 3 groups and tattooed with consecutive numbers. Each group of 20 mice was accommodated in separate cages with ad libitum access to water and food. The animal facility was kept at a 12 h light-dark cycle and the animals were weighted monthly. During the entire study period, the well-being of the animals was monitored daily; mice showing any signs of discomfort, distress or pain, including reduced mobility, inactivity, abnormal posture, lack of grooming, or ulceration exceeding 10 mm in diameter were euthanized. Euthanasia was conducted through cervical dislocation or through asphyxiation with CO_2_. The study followed national guidelines for laboratory animal welfare and was approved by *The Animal Experimental Inspectorate* (J.nr. 2014-15-0201-00096).

### Study Set-Up

Interventions are presented in Table 1. Mice were irradiated with solar simulated UVR 3 times per week during the study period. Five single treatments with IngMeb were given at 4-week intervals (Days 21, 49, 77, 105, 133). Concurrent CP ointment was applied pre and post IngMeb treatment. One week after IngMeb treatment No. 1, 3, and 5 (Days 28, 84, 140), four mice from each group were euthanized and skin biopsies were taken from the backs of the animals. The primary objective was to follow the histological development of UV-damage in the skin on a standardized UV-damage scale. Primary end-point was UV-damage score on Days 28, 84, 140. Biopsies from a control mouse, not receiving UVR were used as a normal skin reference. The secondary objective was to investigate the effect of concurrent CP application on IngMeb-induced LSR. Accordingly, secondary endpoint was LSR-scores during all IngMeb treatments. Mice were followed for up to 140 days.

**Table 1:** Study set-up.

	Group 1 UVR (n = 20)			Group 2 UVR+IngMeb (n = 20)			Group 3 UVR+IngMeb+CP (n = 20)		
Week	UVR	Treatment	Histology	UVR	Treatment	Histology	UVR	Treatment	Histology
**1**	3x3 SED			3x3 SED			3x3 SED		
**2**	3x3 SED			3x3 SED			3x3 SED		
**3**	3x3 SED	-		3x3 SED	Tx1 IngMeb		3x3 SED	Tx1 IngMeb+CP	
**4**	-		4 mice	-		4 mice	-		4 mice
**5**	3x3 SED			3x3 SED			3x3 SED		
**6**	3x3 SED			3x3 SED			3x3 SED		
**7**	3x3 SED	-		3x3 SED	Tx2 IngMeb		3x3 SED	Tx2 IngMeb+CP	
**8**	-			-			-		
**9**	3x3 SED			3x3 SED			3x3 SED		
**10**	3x3 SED			3x3 SED			3x3 SED		
**11**	3x3 SED	-		3x3 SED	Tx3 IngMeb		3x3 SED	Tx3 IngMeb+CP	
**12**	-		4 mice	-		4 mice	-		4 mice
**13**	3x3 SED			3x3 SED			3x3 SED		
**14**	3x3 SED			3x3 SED			3x3 SED		
**15**	3x3 SED	-		3x3 SED	Tx4 IngMeb		3x3 SED	Tx4 IngMeb+CP	
**16**	-			-			-		
**17**	3x3 SED			3x3 SED			3x3 SED		
**18**	3x3 SED			3x3 SED			3x3 SED		
**19**	3x3 SED	-		3x3 SED	Tx5 IngMeb		3x3 SED	Tx5 IngMeb+CP	
**20**	-		4 mice	-		4 mice	-		4 mice

In group 3, CP was applied once daily for five days prior to each IngMeb treatment, 6 h after, and 1-day after IngMeb treatment, in total 7 applications. UVR = ultraviolet radiation, IngMeb = Ingenol Mebutate, CP = clobetasol propionate, SED = standard erythema dose, Tx = Treatment.

### Solar simulated ultraviolet radiation

UVR was given with a UV6 tube (Waldmann, Wheeling, IL, USA) placed between five Bellarium-S SA-1-12 tubes (Wolff System, Atlanta, Georgia, USA) with a maximum wavelength of 365 nm and 5.9% in the UV-B spectrum [19]. UVR was administered as 3 standard erythema doses (SEDs) three times weekly. The UV-dose was measured using a spectroradiometer (Solatell Sola-Hazard 4D Controls Ltd., Cornwall, UK) and UVR-exposure time adjusted continuously to correspond to 3 SED. To allow recovery from IngMeb treatments, each treatment was followed by a one-week pause from UVR.

### Ingenol Mebutate & Clobetasol Propionate

IngMeb-gel (120 μl; Picato^®^ 0.015%, LEO Pharma, Ballerup, Denmark) was applied on the entire dorsal skin of the mice, from neck to tail. CP-ointment (25 μl; Dermovat^®^ 0.05%, GlaxoSmithKline Pharma, Brentford, United Kingdom) was correspondingly applied on the entire dorsal skin of the mice once daily for five days prior to each IngMeb treatment, 6 h after, and 1-day after IngMeb treatment, in total 7 applications.

### Histological preparation

After euthanasia, biopsies from the dorsal skin of the mice were taken at pre-determined positions, fixed in 4% formalin, embedded in paraffin, and cut in 5μm slides. Slides were stained with hematoxylin and eosin for blinded UV-damage evaluation.

### Outcome measurements

#### Histological evaluation of UV-damage

UV-damage evaluations were based on standardized assessments of UV-damage on a ‘UV-damage score scale’. The scale is based on changes known to emerge in hairless mice in response to UVR and have been established previously [20–23]. The scale was tested on histology slides from non-UV-irradiated murine skin and UV-irradiated murine skin before being used for blinded evaluation in the study. The scale evaluates signs of UV-damage in hairless mice, and includes appraisal of stratum corneum, epidermis, and dermis. Assessments were conducted by a blinded dermatopathologist (U.P.) on a categorical scale from 0–3 evaluating (i) keratosis grade of stratum corneum (0 orthokeratotic, 1 focal hyperkeratosis, 2 generalized hyperkeratosis, 3 parakeratosis), (ii) thickness of epidermis (0 3–4 cell layers, 1 5–7 cell layers, 2 8–10 cell layers, 3 10+ cell layers), (iii) dysplasia of epidermis (0 none, 1 present in lower 1/3, 2 present in lower 2/3, 3 present in the entire epidermis), and (iv) dermal actinic damage (0 none, 1 focal, upper papillary layer, 2 generalized, upper papillary layer, 3 reticular layer). The composite UV-score represented the sum of all sub-evaluations (0–12), where higher numbers indicated more severe UV-damage.

#### Local skin responses (LSR)

During all five IngMeb treatments, LSR was registered at baseline and 1-, 2-, 3-, 4-, 5-, 6-, and 7 days after treatment. The LSR evaluation consisted of separate evaluations of erythema, flaking, crusting, vesiculation, bleeding, and ulceration on a scale from 0–4 as described by Rosen at al [16]. In the current study, evaluations of swelling were not conducted but replaced with evaluations of bleeding. The composite LSR-score represented the sum of all sub-evaluations (0–24), where higher numbers indicated more severe skin reactions.

### Statistics

UV-damage score and LSR-responses were compared using unpaired t-test and Spearman correlation was used to evaluate relation between UV-damage and time. P-values were 2-sided and considered statistically significant when less than 0.05. Statistical analyses were performed in SPSS version 23 for Mac (IBM Corporation, Armonk, NY, USA).

## Results

### UV damage

**Fig 1:**
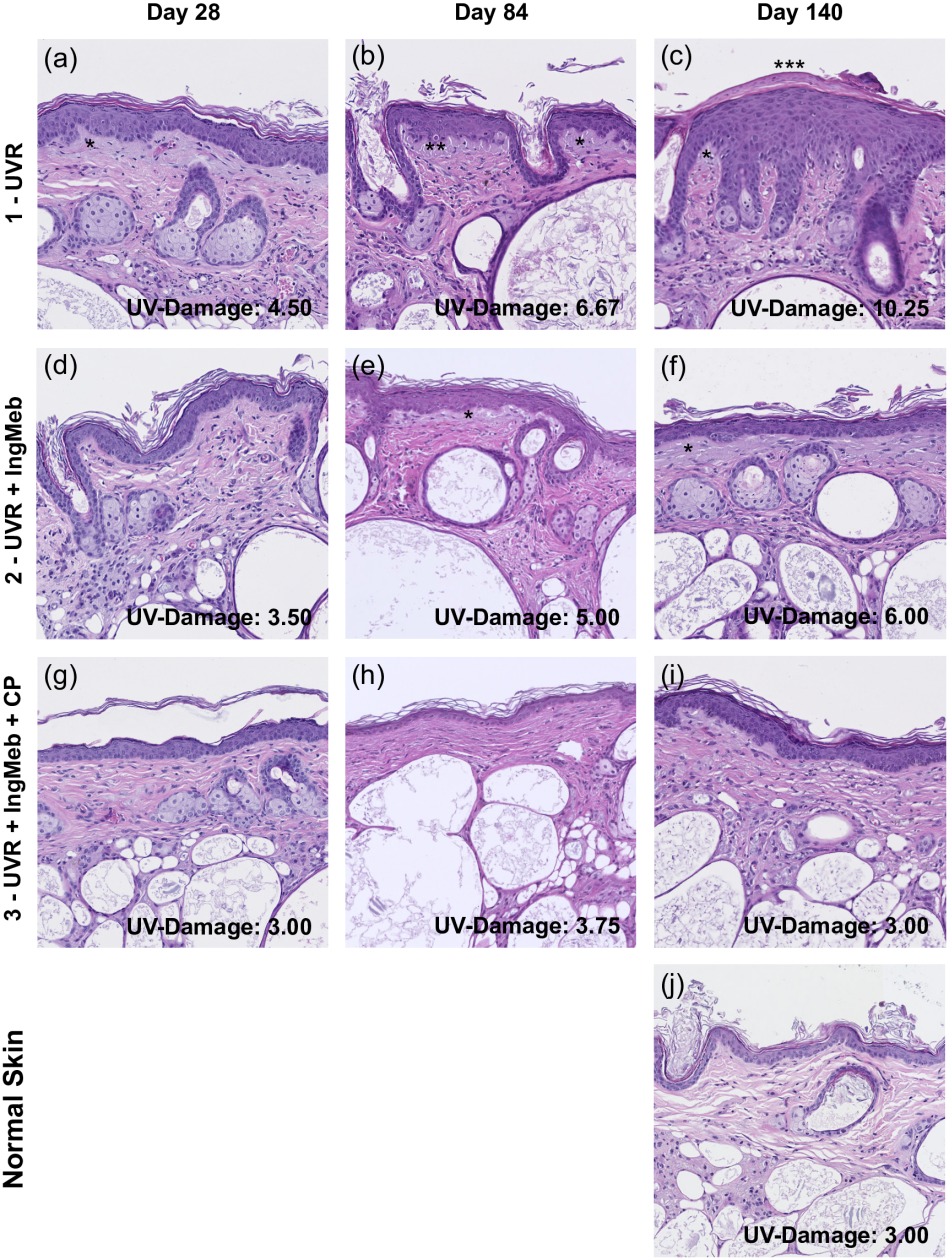
Histology. The figure depicts histological slides from mice exposed to ultraviolet radiation (UVR; a, b and c), 2) UVR and ingenol mebutate (UVR + IngMeb; d, e, and f) 3) UVR, IngMeb and clobetasol propionate (UVR + IngMeb = CP; g, h, and i) and 4) normal skin (j). Histological findings disclose that keratosis grade, epidermal hypertrophy, dysplasia, and actinic dermal damaged increased over time in mice receiving UVR alone (R_s_ = 0.82, p = 0.002), while repeated treatments with ingenol mebutate prevented progression of photodamage (Day 140; UVR 10.25 vs. UVR+IngMeb 6.00, p = 0.002). Concurrent treatments with CP potentiated the prophylactic effect of IngMeb with UV-damage scores similar to normal skin at day 140 (UVR+IngMeb+CP 3.00, Normal skin 3.00). * dermal actinic damage; ** dysplasia present in lower 2/3 of epidermis and an absent basal layer with the presence of a Zytoid body; *** parakeratosis.

In mice receiving UVR alone, keratosis grade, epidermal hypertrophy, dysplasia, and actinic dermal damage increased over time (Table 2, Fig 1); composite UV-damage score at day 28 was 4.50, which increased to 6.67 at day 84, and 10.25 at day 140 (R_s_ = 0.82, p = 0.002). IngMeb treatments prevented progression of photodamage in terms of keratosis grade, epidermal hypertrophy, dysplasia, and dermal actinic damage; composite UV-damage score was 3.50, 5.00, and 6.00 at day 28, 84, and 140, respectively, (UVR vs. UVR+IngMeb day140, p = 0.002; Table 2, Fig 1).

**Table 2:** Ultraviolet damage scores 1 week after ingenol mebutate treatment No. 1, 3 and 5.

	Day 28 UV-damage score	Day 84 UV-damage score	Day 140 UV-damage score
**Group 1 –UVR** (R_s_ = 0.82, p = 0.002*)	**4.50**	**6.67**	**10.25**
Keratosis grade	1.00	1.67	2.75
Epidermal thickness	0.75	1.67	2.50
Dysplasia	1.25	1.33	2.25
Dermal actinic damage	1.50	2.00	2.75
**Group 2—UVR + IngMeb** (R_s_ = 0.70, p = 0.011*)	**3.50**	**5.00**	**6.00**
Keratosis grade	0.75	1.0	1.75
Epidermal thickness	0.75	1.0	1.00
Dysplasia	1.25	1.5	1.00
Dermal actinic damage	0.75	1.5	2.25
**Group 3—UVR+ IngMeb+CP** (R_s_ = -0.03, p = 0.936*	**3.00**	**3.75**	**3.00**
Keratosis grade	1.0	0.75	0.00
Epidermal thickness	0.50	0.75	0.67
Dysplasia	0.75	1.00	1.00
Dermal actinic damage	0.50	1.25	1.33
p-value, UVR vs. UVR+IngMeb:	0.600	0,310	0.002**
p-value, UVR vs. UVR+IngMeb+CP:	0.487	0,118	<0.001**
p-value, UVR+IngMeb vs. UVR+IngMeb+CP:	0.760	0,235	0.014**

UVR = ultraviolet radiation, IngMeb = Ingenol Mebutate, CP = clobetasol propionate

*Spearman Correlation, UV-damage/time.

** Statistically significant.

Concurrent CP applications potentiated the UV-protective effect of IngMeb (Table 2, Fig 1). No progression in photodamage was observed during the 140 days of UVR exposure (R_s_ = -0.03, p = 0.936). Composite UV-damage score was 3.00 at day 28, 3.75 at day 84, and 3.00 at day 140, reaching statistical significance at day 140 (UVR vs. UVR+IngMeb+CP, p = <0.001; UVR+IngMeb vs. UVR+IngMeb+CP, p = 0.014).

### Local skin responses

**Fig 2:**
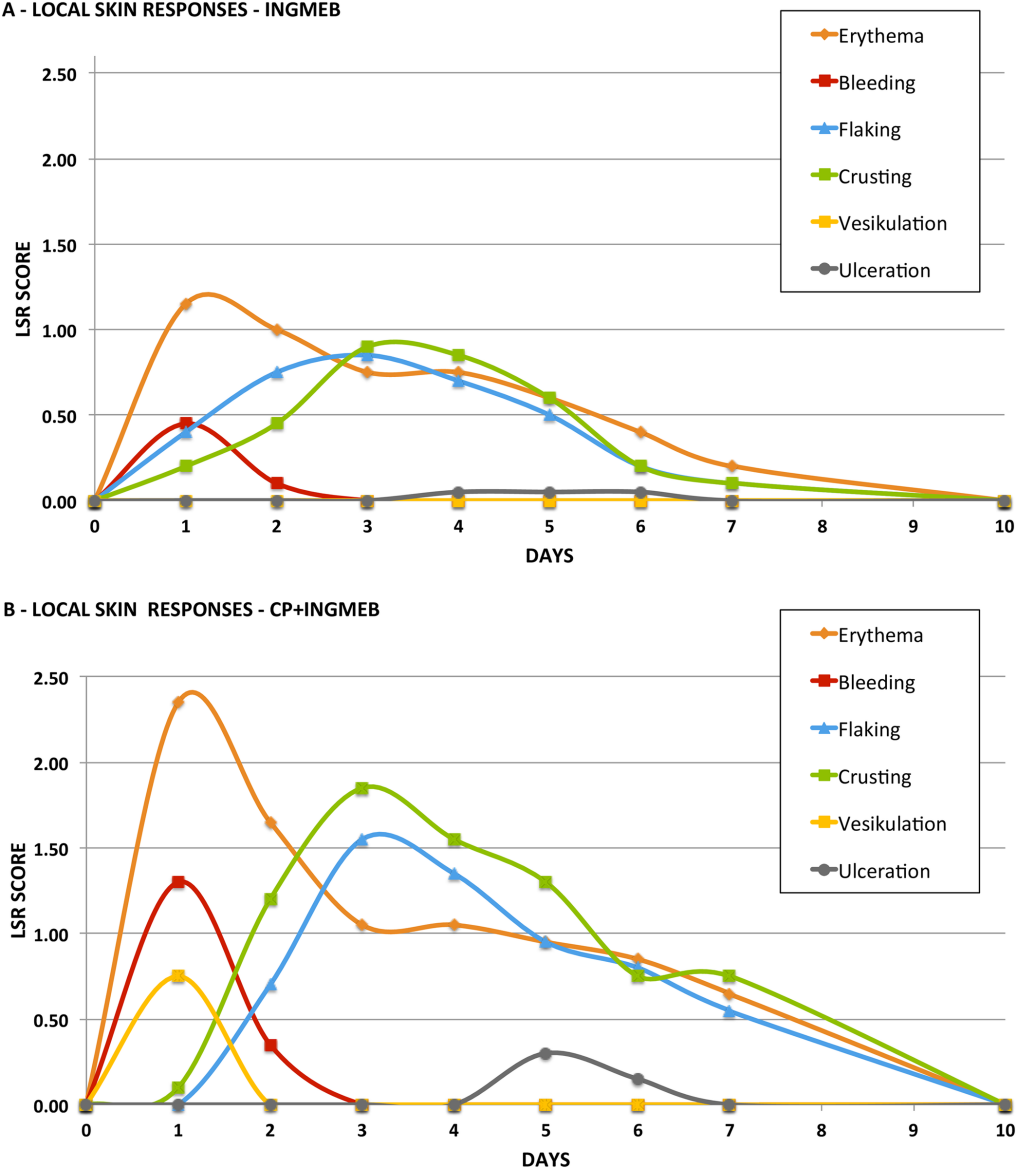
Local skin responses. The figure depicts the evolution of individual local skin responses (LSR) in mice treated with ingenol mebutate alone (IngMeb; A) and concurrent clobetasol (IngMeb+CP: B). Erythema, bleeding, and vesiculation developed rapidly after IngMeb application peaking on day 1. Flaking and crusting emerged on day 2, culminating on day 3 while ulceration had a delayed onset and reached peak intensity on day 5. Concurrent CP application did not alleviate the LSR but exacerbated all individual responses, including erythema, bleeding, vesiculation, flaking, crusting and ulceration. The skin was normalized by day 10 post-treatment in both IngMeb and IngMeb+CP treated mice.

LSR included erythema, flaking, crusting, bleeding, vesiculation, and ulceration. Maximal composite LSRs-score were of moderate intensity in mice treated with IngMeb alone (max LSR treatment 1–5: 3.6–5.5; Table 3), while concurrent treatment with CP increased LSR significantly during IngMeb in three out of five treatments (Table 3, Fig 2). CP increased the prevalence of intracutaneous bleedings in treatment 1 and 2 (p < 0.037) and in areas where such bleedings occurred, wounds with crusting, flaking, and subsequently ulceration appeared.

**Table 3:** Maximum local skin responses (LSR) in areas treated with IngMeb and IngMeb+CP.

	Treatment 1 LSR	Treatment 2 LSR	Treatment 3 LSR	Treatment 4 LSR	Treatment 5 LSR
**Group 2—UVR + IngMeb**	**3.0**	**2.8**	**3.6**	**2.6**	**4.2**
Erythema	1,2	1,0	1,0	1,0	1,5
Flaking	1,0	0,9	1,2	0,9	1,5
Crusting	0,9	0,9	1,4	0,9	1,0
Pustulation	0,0	0,0	0,1	0,0	0,3
Ulceration	0,1	0,2	0,0	0,3	0,8
Bleeding	0,1	0,2	0,0	0,3	0,8
**Group 3—UVR+ IngMeb+CP**	**5.2**	**5.4**	**3.6**	**3.8**	**5.5**
Erythema	2,4	1,3	1,0	1,0	2,0
Flaking	1,7	1,9	1,3	1,4	1,5
Crusting	1,9	2,1	1,3	1,1	1,8
Pustulation	0,8	0,3	0,1	0,5	1,0
Ulceration	0,6	0,9	0,0	0,1	0,8
Bleeding	0,6	0,9	0,0	0,1	0,8
Effect of CP: P-value, comparing UVR+IngMeb vs. UVR+IngMeb = CP	<0.001*	<0.001*	0.886	0.015*	0.376

UVR = ultraviolet radiation, IngMeb = Ingenol Mebutate, CP = clobetasol propionate,

*Statistically significant.

Evolution of individual responses during first IngMeb treatment is presented in Fig 2; erythema, bleeding, and vesiculation developed rapidly after IngMeb application peaking on day 1. Flaking and crusting emerged on day 2, culminating on day 3 while ulceration had a delayed onset and reached peak intensity on day 5. The skin normalized by day 10 post-treatment.

## Discussion

This study is the first to demonstrate that repeated field-directed treatments with IngMeb prevent progression of cutaneous photodamage in hairless mice, suggesting that IngMeb may serve as prophylactic treatment for UV-induced tumors.

Incidence rates of NMSC in Europe have increased steadily since the 1960s and more than tripled over the last 50 years [1]. As UVR cannot be completely avoided, chemopreventative treatments are needed to complement primary skin cancer prevention. Various systemic and topical formulations have been studied for this purpose, including retinoids, non-steroidal anti-inflammatory drugs, nicotinamide and difluoromethylornithine, but the necessity of daily use render them cumbersome in practice. Herein, we demonstrate that monthly treatments with IngMeb prevent progression of photodamage, including development of hyperkeratosis, epidermal hypertrophy, dysplasia, and dermal actinic damage. In another murine study, such chemoprophylaxis was shown to postpone tumor formation (from 168 to 189; p = 0.025), suggesting that prevention of early actinic damage may translate into effective prophylactic treatments for UV-induced tumors [24]. Additionally, the study substantiates the evidence of IngMeb as a field-directed treatment, not only targeting pre-existing AKs but preventing progression of UV-damage in surrounding pre-cancerous skin. A third murine study investigating IngMeb treatment of pre-cancerous skin, showed a 70% reduction in tumor formation [15]. The authors argued that IngMeb-induced necrosis and apoptosis of the epidermis and subsequent re-epithelialization with non-irradiated keratinocytes from bordering hair follicles was the major mechanism behind the prophylactic effect. However, previous murine studies conducted in C3.Cg/TifBomTac species have suggested that murine tumors often originate from hair follicles [25]. In addition to epidermal renewal, IngMeb also activates Protein Kinase C -delta (PKC-δ) which has been found to induce apoptosis in cancer cell lines [10,26] and induce upregulation of neutrophil mediators, (IL-8, TNF-α, ICAM-1, and E-selectin), resulting in massive neutrophil invasion to the skin [27]. Recent investigations have demonstrated that the immune infiltration is not confined to the epidermis, but reaches deep into the hair follicles and IngMeb may thus be able to eradicate aberrant cells in profound parts of their structure. Through a combination of epidermal renewal and immune activation, repeated treatments with IngMeb are believed to provide the observed photoprevention as demonstrated herein.

This study is the first to document the evolution of LSR after a single IngMeb treatment, finding that the intensity of individual responses was time-dependent. Vesiculation and bleeding appeared immediately after IngMeb application. At cessation of bleeding and vesiculation, flaking and crusting emerged and cumulated before ulceration peaked. Erythema was present from day 1 and decreased gradually until the skin normalization by day 10. Observed reactions may be too severe to justify prophylactic use, and in order for IngMeb to gain clinical impact as a prophylactic remedy, the LSR must be kept at a minimum. Previous studies have demonstrated that corticosteroids can block IngMeb-induced neutrophil invasion, and concurrent CP was thus believed to alleviate LSR[18]. Contrary to our hypothesis, CP failed to alleviate LSR and in contrast generated more severe LSR. Murine studies have previously argued that if IngMeb comes in contact with the dermal capillary plexus, disruption of the vasculature may cause intracutaneous bleeding [13,18]. In mice treated with IngMeb, bleeding was not common, but CP increased the prevalence of intracutaneous bleedings and in areas where such bleedings occurred, wounds with crusting and flaking appeared. Since murine epidermis is only 3-cell layer thick, the pre-treatment with CP may have disrupted the skin barrier and allowed IngMeb to come in contact with the dermal vasculature. Alternatively, the application of CP-ointment 6h after IngMeb may have had an occlusive effect, which again is known to increase cutaneous uptake and may have caused the observed bleedings. The LSR are thus likely increased in CP-treated mice due to a deeper penetration of IngMeb; CP’s effect on inflammation is in this instance unclear, as a potential block of neutrophil invasion is likely overshadowed by the increased LSR caused by the intracutaneous bleeding. Additionally, a recent clinical trial examined CP effect on IngMeb-induced inflammation after three daily applications of IngMeb in patients with AKs [28]; the study found no alleviating effect of CP, and it is thus feasible that CP has no impact on IngMeb-induced LSR. However, in the clinical trial, CP was applied after finalized IngMeb treatment and earlier initiation of CP applications needs to be investigated before final conclusions can be drawn.

The hairless mouse model is widely employed for investigations of photocarcinogenesis [29]. The C3.Cg/TifBomTac mice have a very low incidence of spontaneous skin tumors and their response to UVR is similar to humans, developing squamous cell carcinomas and having the ability to tan. Still, major morphological differences between mouse and human skin render murine skin more permeable to most topical agents, and findings regarding prevention of photodamage, LSR, and effects of CP cannot be directly extrapolated to humans. Additionally, prevention of photodamage is not equivalent to NMSC prophylaxis; the impact of photodamage prevention on postponement of cutaneous cancers remains to be investigated.

In conclusion, repeated field-directed treatments with IngMeb prevent progression of cutaneous photodamage in hairless mice, while CP cannot be used to alleviate IngMeb-induced LSR. The findings suggest that IngMeb may potentially serve as a prophylactic treatment for UV-induced BCC and SCC.

## Supporting Information

S1 File Database with raw data. (XLSX)
